# Prospective observational study quantifying maternal-fetal fentanyl transmission in epidural analgesia infusion using umbilical cord blood and neonatal meconium

**DOI:** 10.1038/s41372-025-02416-9

**Published:** 2025-09-18

**Authors:** Nanyaly M. Santiago-Aponte, Dongxiao Sun, Tonya S. King, Tammy E. Corr

**Affiliations:** 1 Penn State Milton S. Hershey Medical Center Children’s Hospital, Hershey, PA USA; 2 Penn State College of Medicine, Department of Pediatrics, Division of Neonatal-Perinatal Medicine, Hershey, PA USA; 3 Penn State College of Medicine, Department of Pharmacology, Mass Spectrometry Core Facilities, Hershey, PA USA; 4 Penn State College of Medicine, Department of Public Health Sciences, Hershey, PA USA

**Keywords:** Paediatrics, Diagnostic markers

## Abstract

**Objective:**

To determine whether fetal exposure to maternal epidural fentanyl can result in a positive meconium drug test.

**Study design:**

Quantitative evaluation of fentanyl levels in cord blood and meconium of infants ≥37 weeks whose mothers received epidural analgesia was performed using High Performance Liquid Chromatography-tandem Mass Spectrometry. The association between dose and duration of maternal epidural and fentanyl levels in cord blood and meconium was evaluated.

**Results:**

In 298 mother-infant dyads, median duration of fentanyl epidural was 6.8 hours. There was a strong positive correlation between epidural duration and fentanyl levels in meconium (Spearman rho = 0.70, 95%CI [0.64-0.76]), *p* < 0.001). Positive predictive value for fentanyl detection in meconium >0.05 ng/g for epidural exposure of >4 hours was 93.9%.

**Conclusion:**

There is a strong positive correlation between maternal epidural duration and fentanyl in meconium. Providers can anticipate a positive meconium drug screen for fentanyl in the setting of a maternal epidural.

## Introduction

The opioid epidemic in the United States remains a public health problem [1–3], and maternal opioid use now affects 8.2 out of 1000 hospital deliveries [2]. The American College of Obstetricians and Gynecologists (ACOG) recommends universal screening of pregnant individuals for substance use disorders with subsequent maternal and newborn drug testing based on results of risk assessment and with informed maternal consent [4, 5].

Given both the greater illicit use of fentanyl as well as the increased contamination of opioid drug supplies with fentanyl [6, 7], some birthing centers have expanded neonatal meconium drug testing to include fentanyl. However, interpretation of these drug results can be challenging as fentanyl is a frequently used medication for pain control in the healthcare setting [8], particularly during labor. Furthermore, in cases where maternal drug testing may be available for review, fentanyl is not always specifically tested for, further complicating interpretation of these results.

The current standard of care for adequate management of peripartum pain are epidural infusions containing a local anesthetic (bupivacaine or ropivacaine) and an opioid such as fentanyl or sufentanil [9, 10]. As such, the majority of hospitals across the U.S. provide local anesthetic epidural infusions with added fentanyl to as many as 70% of laboring mothers [1, 9]. Despite its widespread use and a theoretical risk of fentanyl transmission to the fetus, there are limited studies that have assessed the relationship between maternal epidural fentanyl administration and fetal accumulation, and there are no data on fentanyl’s ability to contaminate newborn meconium [11–13]. Establishing the relationship between transmission of fentanyl via intrapartum maternal epidural and its accumulation in the fetus may provide insight into opioid exposure, which has important clinical implications regarding the postnatal management of the neonate with a fentanyl-positive meconium test.

This study aims to determine whether fetal exposure to maternal fentanyl, delivered via epidural analgesia during labor, can result in a positive meconium drug test for fentanyl in newborn infants, and if so, to determine whether a relationship exists between fentanyl dose delivered via maternal epidural infusion and fetal accumulation by quantitatively evaluating levels of fentanyl in umbilical cord blood and meconium. We hypothesized that exposure to fentanyl via maternal epidural analgesia will result in a positive meconium for fentanyl.

## Methods

This single center, prospective, observational study was performed at Penn State Milton S. Hershey Medical Center at the Children’s Hospital and Women and Babies Center. Penn State Medical Center is tertiary care, academic institution with approximately 2300 deliveries per year, a 56-bed level IV Neonatal Intensive Care Unit (NICU), and an active Maternal-Fetal Medicine program. All human participants gave written, informed consent. Subjects were enrolled from January 2023 to January 2024. The study was approved by Penn State’s Institutional Review Board, STUDY00020718.

### Patient population

Male and female infants of ≥ 37 weeks of gestation at birth whose mothers received a continuous bupivacaine and fentanyl epidural infusion during labor were included in the study. Three mother-infant dyads served as control subjects for the development of the High Performance Liquid Chromatography-tandem Mass Spectrometry (HPLC-MS) protocol for meconium, and these mothers did not receive intrapartum fentanyl epidural. The remainder of the maternal patients received an initial local anesthetic plus a fentanyl bolus, typically ranging from 0 to 10 mcg, followed by a continuous epidural infusion of bupivacaine 1.25 mg/ml + fentanyl 2 mcg/ml and intermittent boluses through Patient Controlled Analgesia (PCA) pumps. A convenience sample of eligible mothers who denied fentanyl use in any form (intravenous, intramuscular, transdermal, or intranasal) in the second or third trimester of pregnancy (>14 weeks gestation) were approached and consented. Infants with neurologic or metabolic injury or symptoms that could interfere with or mimic signs of opioid withdrawal such as hypoxic ischemic encephalopathy, neonatal ischemic or hemorrhagic stroke, neonatal seizures, uncontrolled hypoglycemia, or known inborn errors of metabolism, major congenital anomalies, known genetic abnormalities or delayed passage of meconium requiring contrast enema evaluation were excluded from participation in the study. In addition, infants who received therapeutic fentanyl in the neonatal intensive care unit before the meconium specimen was collected or whose mothers admitted to or had medical record evidence of fentanyl use during the second or third trimesters of pregnancy or positive urine drug screen (if obtained per clinical judgement) for opioids on admission were also excluded. Only English and Spanish speaking subjects were enrolled in the study.

### Demographic and health data collection

Demographic and pregnancy health data were collected via electronic medical record review as well as a 19-item online questionnaire. Mothers received the questionnaire in their preferred language, English or Spanish, upon enrollment. Epidural infusion data such as duration of epidural and total dose of fentanyl in epidural was collected via electronic medical record review. Study data were managed using Research Electronic Data Capture (REDCap) tools hosted at Penn State Health Milton S. Hershey Medical Center and Penn State College of Medicine.

### Umbilical cord blood and meconium sample acquisition and preparation

Umbilical cord blood samples of mixed arterial and venous blood were collected in EDTA-treated lavender top tubes at the time of delivery in accordance with Penn State Milton S. Hershey Medical Center’s delivery protocol. Umbilical cord blood samples were stored in a 4.5 ^ο^C refrigerator until ready for processing. Umbilical cord blood samples were then centrifuged for 20 min at 2000 rpm at a temperature of 4 ^ο^C to obtain plasma. Meconium samples for infants were collected during the first 48 h of life and placed in a sterile cup. Plasma samples and meconium samples were stored in −80 ^ο^C freezer. All samples were processed by Sciex High Performance Liquid Chromatography with QTRAP 6500 + Mass Spectrometry system, a methodology used for screening and confirmatory testing of drug metabolites in meconium due to its sensitive and specific results [14–16]. The lowest capable limit of fentanyl detection in meconium using this method was 0.01 ng/g.

### Statistical analysis

A sample size of 246 was required for a level of precision of ±0.10 (width = 0.20) of a 95% confidence interval around an estimate of Spearman rank correlation for the association of maternal epidural exposure and fentanyl concentration in the meconium. Demographic information was summarized using frequencies and percentages. The associations between duration and dose of maternal epidural infusion and the fentanyl concentration in the infant’s cord blood and meconium were evaluated by estimating the Spearman rank correlation coefficient. Area under the curve (AUC) of the receiver operating characteristic curve for maternal epidural exposure as a predictor of fentanyl in the meconium >0.05 ng/g, a concentration based on commercial testing standards for positive fentanyl results, was estimated using logistic regression. Sensitivity, specificity, positive predictive value, and negative predictive value were estimated for different thresholds of maternal epidural exposure. Significance was defined as *p* < 0.05, and statistical analyses were performed using SAS statistical software version 9.4 (SAS Institute Inc., Cary NC).

## Results

**Fig. 1:**
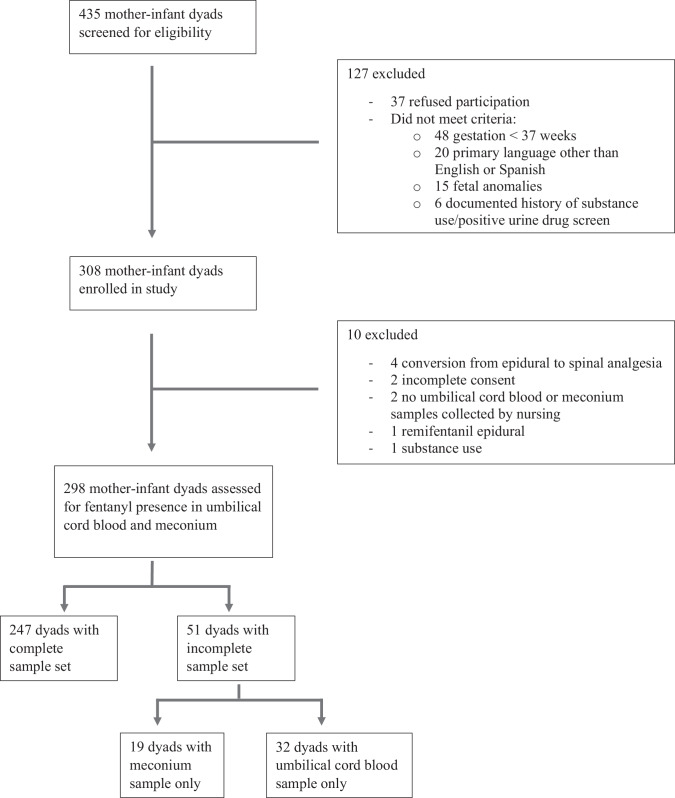
Flow diagram for subject selection and enrollment.

A total of 308 eligible subjects were enrolled in the study upon admission to labor and delivery or 24 h post-partum (Fig. 1). All pregnancies were singletons with the exception of one set of monochorionic-diamniotic twins and one set of dichorionic-diamniotic twins. All enrolled mothers denied history of prescribed or unprescribed use of fentanyl during the second and third trimester of the current pregnancy and had healthy pregnancies with early initiation of prenatal care (Table 1).

**Table 1:** Maternal characteristics of study participants based on self-reported maternal questionnaire.

Characteristic	*n* (%)
**Age**	
18–29 years old	92 (45.3)
30–39 years old	105 (51.7)
40–49 years old	5 (2.5)
Prefer not to answer	1 (0.5)
**Medical conditions**	
Diabetes	6 (2.0)
High blood pressure	23 (7.7)
Chronic pain	3 (1.0)
**Start of prenatal care**	
First trimester	192 (96.5)
Second trimester	4 (2.0)
Third trimester	1 (0.5)
Did not have prenatal care	2 (1.0)
**Alcohol consumption**	
Never	109 (54.2)
Monthly or less	60 (29.9)
2–4 times a month	27 (13.4)
2–3 times a week	5 (2.5)
4 or more times a week	0 (0)
**Smoking history**	
Never	197 (98)
Once a week or less	1 (0.5)
Once a day	1 (0.5)
Several times a day	2 (1.0)

**Fig. 2:**
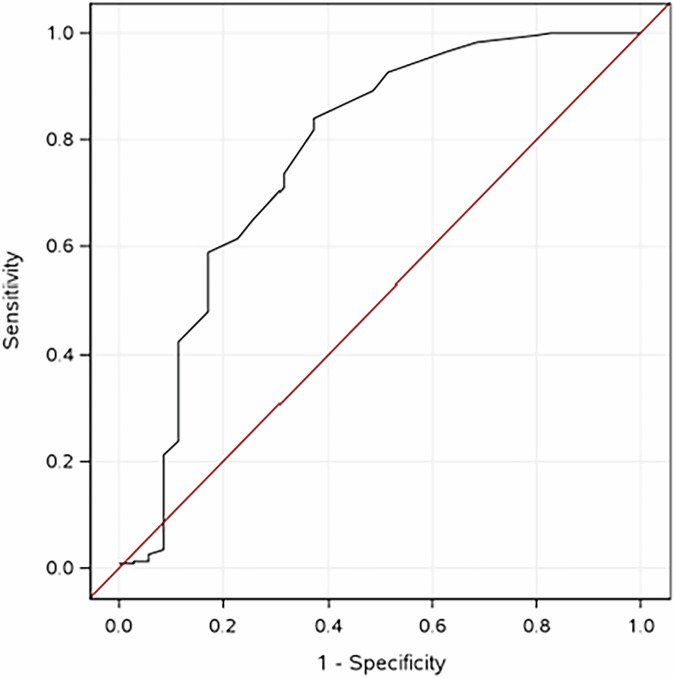
Maternal epidural exposure predicts presence of fentanyl in meconium at concentrations >0.05 ng/g (AUC 0.77, 95% CI 0.67–0.87, *p* < 0.001).

**Fig. 3:**
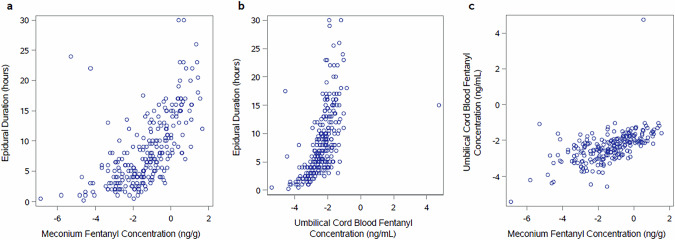
Epidural duration correlates with fentanyl concentrations in meconium and umbilical cord blood. **a** Fentanyl concentrations in meconium by epidural duration. **b** Fentanyl concentrations in umbilical cord blood by epidural duration. **c** Fentanyl concentrations in meconium by fentanyl concentrations in umbilical cord blood. All fentanyl concentrations are reported as the natural-log transformed concentrations.

Intrapartum bupivacaine-fentanyl epidural duration was widespread, ranging from 1 to 30 h. The median duration of maternal, intrapartum fentanyl epidural infusion was 6.8 hours (Q1,Q3 4.0,11.5), and a median of 152.3 mcg of fentanyl was delivered (Q1,Q3 86.6, 268.0). Fentanyl in the meconium >0.05 ng/g was detected in 231 (86.8%) of the 266 meconium samples with available data. Maternal epidural exposure predicted the presence of fentanyl in meconium at concentrations >0.05 ng/g (AUC 0.77, 95% CI 0.67–0.87, *p* < 0.001) (Fig. 2). There was a strong positive correlation between maternal epidural infusion duration and dose and fentanyl levels in the meconium ((Spearman rho = 0.74, 95% CI [0.68–0.79]), *p* < 0.001 for dose) and (Spearman rho = 0.70, 95%CI [0.64–0.76]), *p* < 0.001 for duration (Fig. 3a). There was also a strong positive correlation between the duration of maternal epidural infusion and fentanyl concentrations in umbilical cord blood (Spearman rho = 0.72, 95%CI [0.66–0.78]), *p* < 0.001 (Fig. 3b). Finally, there was a moderate positive correlation between the concentration of fentanyl in umbilical cord blood and the concentration of fentanyl in meconium (Spearman rho = 0.64, 95%CI [0.55–0.70]), *p* < 0.001 (Fig. 3c). The sensitivity and specificity of intrapartum epidural exposure of >4 h as a predictor of fentanyl detection in meconium >0.05 ng/g were 73.6% and 68.6%, respectively, with 93.9% positive predictive value and 28.2% negative predictive value (Table S1).

## Discussion

This single center, prospective, observational study found that there is a strong positive correlation between maternal epidural infusion duration and fentanyl detection in newborn umbilical cord blood and meconium. To our knowledge, this is the first study that has evaluated the ability of fentanyl from a maternal epidural infusion to be quantified in both the umbilical cord blood and meconium of an exposed newborn. While prior studies have looked for the presence of fentanyl in urine of infants whose mothers received intrapartum epidural infusions [17, 18], these studies have limited value in the clinical setting as newborn urine toxicology is inadequate for assessing prenatal drug exposure and is reflective only of prenatal drug exposure in the last 48 h. Conversely, meconium samples for toxicology testing are easy to collect, are typically readily available at the time an infant may begin to display symptoms concerning for opioid withdrawal and allow for the detection of prenatal drug exposure as early as 18 weeks of gestation. These features make meconium an ideal test to screen for prenatal drug exposure.

Fentanyl has positioned itself as the leading opioid in the illicit drug market due to its versatility and availability [6, 7]. As there is no ability to differentiate illicitly-manufactured fentanyl and its metabolites in a meconium toxicology test, it is important to understand whether fentanyl exposure resulting from intrapartum pain control can be detected in meconium for both maternal and infant wellbeing and health. While there are currently mechanisms in place to screen at-risk pregnancies such as the Screening Brief Intervention and Referral to Treatment tool [19], self-reporting, and universal screening [16], these screens may trigger meconium toxicology testing at delivery, which sometimes result in unanticipated findings of fentanyl detection.

There is an array of harmful consequences that arise from misinterpretation of a drug test. A positive newborn drug test is fraught with psychological, social, and legal ramifications for the mother, and it can be a catalyst for maternal labeling and stigma, separation from her infant, and referral to Children and Youth Services [20]. Mothers with opioid use disorder (OUD), or perceived to have OUD, report experiencing negative interactions with healthcare providers, judgmental attitudes, questioning of parental skills and unnecessary testing [21], uncovering stereotyping and implicit bias held by providers [22–28]. Beyond Children and Youth Services involvement, concern for illicit opioid use during pregnancy can lead to potential loss of child custody and, in some states, charges of child abuse or civil commitment [20, 29, 30]. Thus, understanding rates of fentanyl transmission from the maternal epidural space to fetal meconium is imperative.

Using the same testing (HPLC-MS) that is used to detect fentanyl in commercial laboratories used for the clinical setting, our results show that measurable fentanyl levels were present in both umbilical cord blood and meconium of all newborns whose mothers received an intrapartum fentanyl epidural infusion, suggesting that there is, indeed, transfer of fentanyl from the maternal epidural space to the maternal circulation and, ultimately, into fetal circulation. These results support the idea that maternal exposure to fentanyl via an epidural infusion is sufficient to result in measurable fentanyl levels in neonates and can help clarify concerns regarding a positive fentanyl meconium toxicology within a clinical setting.

One limitation to this study is that maternal drug testing was not performed prior to delivery to confirm absence of fentanyl exposure during pregnancy. However, given the known procedural collection of infant meconium for fentanyl drug testing, it is unlikely that a pregnant person with an active fentanyl use disorder would volunteer to participate in the study. Despite this limitation, we have established a clear connection between maternal epidural infusions and newborn meconium toxicology screens, and we anticipate the results of this study will provide clinically useful knowledge for pediatric, obstetric, and anesthesia providers who care for pregnant persons and their newborns as well as the social, legal, and community systems that interact with mother-infant dyads affected by OUD.

## Conclusion

Maternal epidural fentanyl infusion is associated with quantifiable levels of fentanyl in newborn umbilical cord blood and meconium. These results should prompt clinical providers to anticipate a positive meconium drug screen for fentanyl in newborns in the setting of a maternal intrapartum epidural infusion.

## Supplementary information

Supplemental Table 1

## Data Availability

Deidentified data sets are available upon request of the authors.

## References

[1] Haight SC, Ko JY, Tong VT, Bohm MK, Callaghan WM. Opioid use disorder documented at delivery hospitalization - United States, 1999-2014. MMWR Morb Mortal Wkly Rep. 2018;67:845–9. 30091969 10.15585/mmwr.mm6731a1 PMC6089335

[2] Hirai AH, Ko JY, Owens PL, Stocks C, Patrick SW. Neonatal abstinence syndrome and maternal opioid-related diagnoses in the US, 2010-2017. Jama 2021;325:146–55. 33433576 10.1001/jama.2020.24991 PMC7804920

[3] Ko JY, D’Angelo DV, Haight SC, Morrow B, Cox S, Salvesen von Essen B, et al. *Vital Signs:* Prescription Opioid Pain Reliever Use During Pregnancy — 34 U.S. Jurisdictions, 2019. MMWR Morb Mortal Wkly Rep. 2020;69:897–903. 32673301 10.15585/mmwr.mm6928a1 PMC7366850

[4] Committee Opinion No. 711: Opioid Use and Opioid Use Disorder in Pregnancy. Obstet Gynecol. 2017;130:e81–94. 28742676 10.1097/AOG.0000000000002235

[5] Patrick SW, Barfield WD, Poindexter BB. Committee on fetus and newborn, committee on substance use and prevention. Neonatal Opioid Withdrawal Syndrome. Pediatrics 2020;146:e2020029074. 33106341 10.1542/peds.2020-029074

[6] Park JN, Rashidi E, Foti K, Zoorob M, Sherman S, Alexander GC. Fentanyl and fentanyl analogs in the illicit stimulant supply: results from U.S. drug seizure data, 2011-2016. Drug Alcohol Depend. 2021;218:108416. 33278761 10.1016/j.drugalcdep.2020.108416 PMC7751390

[7] Rosenblum D, Unick J, Ciccarone D. The rapidly changing US illicit drug market and the potential for an improved early warning system: evidence from Ohio drug crime labs. Drug Alcohol Depend. 2020;208:107779. 31931266 10.1016/j.drugalcdep.2019.107779 PMC7096152

[8] Callahan EC, Lim S, George RB. Neuraxial labor analgesia: Maintenance techniques. Best Pr Res Clin Anaesthesiol. 2022;36:17–30. 10.1016/j.bpa.2022.03.001 35659953

[9] Nanji JA, Carvalho B. Modern techniques to optimize neuraxial labor analgesia. Anesth Pain Med. 2018;13:233–40.

[10] Hussain N, Lagnese CM, Hayes B, Kumar N, Weaver TE, Essandoh MK, et al. Comparative analgesic efficacy and safety of intermittent local anaesthetic epidural bolus for labour: a systematic review and meta-analysis. Br J Anaesth. 2020;125:560–79. 32703549 10.1016/j.bja.2020.05.060

[11] Salameh KM, Anvar Paraparambil V, Sarfrazul A, Lina Hussain H, Sajid Thyvilayil S, Samer Mahmoud A. Effects of labor epidural analgesia on short term neonatal morbidity. Int J Women’s Health. 2020;12:59–70. 32099485 10.2147/IJWH.S228738 PMC7007791

[12] Beilin Y, Bodian CA, Weiser J, Hossain S, Arnold I, Feierman DE, et al. Effect of labor epidural analgesia with and without fentanyl on infant breast-feeding: a prospective, randomized, double-blind study. Anesthesiology 2005;103:1211–7. 16306734 10.1097/00000542-200512000-00016

[13] Wang K, Cao L, Deng Q, Sun LQ, Gu TY, Song J, et al. The effects of epidural/spinal opioids in labour analgesia on neonatal outcomes: a meta-analysis of randomized controlled trials. Can J Anaesth. 2014;61:695–709. 25011701 10.1007/s12630-014-0185-y

[14] Wright TE. Biochemical screening for in utero drug exposure. Drug Metab Lett. 2015;9:65–71. 26411469 10.2174/1872312809666150904182334

[15] Hernandez A, Lacroze V, Doudka N, Becam J, Pourriere-Fabiani C, Lacarelle B, et al. Determination of prenatal substance exposure using meconium and orbitrap mass spectrometry. Toxics. 2022;10:55. 35202242 10.3390/toxics10020055 PMC8875502

[16] Price HR, Collier AC, Wright TE. Screening pregnant women and their neonates for illicit drug use: consideration of the integrated technical, medical, ethical, legal, and social issues. Front Pharm. 2018;9:961. 10.3389/fphar.2018.00961 PMC6120972 30210343

[17] Novikov N, Melanson SEF, Ransohoff JR, Petrides AK. Rates of fentanyl positivity in neonatal urine following maternal analgesia during labor and delivery. J Appl Lab Med. 2020;5:686–94. 32603437 10.1093/jalm/jfaa027

[18] Siegel MR, Mahowald GK, Uljon SN, James K, Leffert L, Sullivan MW, et al. Fentanyl in the labor epidural impacts the results of intrapartum and postpartum maternal and neonatal toxicology tests. Am J Obstet Gynecol. 2023;228:741. 10.1016/j.ajog.2022.11.1293 36427599

[19] Screening, Brief Intervention and Referral to Treatment (SBIRT). Rockville, MD: Substance Abuse and Mental Health Services Administration, August 2022. (https://www.samhsa.gov/sbirt).

[20] Sutter MB, Gopman S, Leeman L. Patient-centered care to address barriers for pregnant women with opioid dependence. Obstet Gynecol Clin North Am. 2017;44:95–107. 28160896 10.1016/j.ogc.2016.11.004

[21] Perlman NC, Cantonwine DE, Smith NA. Racial differences in indications for obstetrical toxicology testing and relationship of indications to test results. Am J Obstet Gynecol MFM. 2022;4:100453. 34352428 10.1016/j.ajogmf.2021.100453

[22] Santoro TN, Santoro JD. Racial bias in the US opioid epidemic: a review of the history of systemic bias and implications for care. Cureus 2018;10:e3733. 30800543 10.7759/cureus.3733 PMC6384031

[23] Earnshaw V, Smith L, Copenhaver M. Drug addiction stigma in the context of methadone maintenance therapy: an investigation into understudied sources of stigma. Int J Ment Health Addict. 2013;11:110–22. 23956702 10.1007/s11469-012-9402-5 PMC3743126

[24] Short VL, Alexander K, Gannon M, Abatemarco DJ, Goyal NK. What aspects of their child’s primary care do mothers value? A qualitative analysis of perspectives of women in treatment for opioid use disorder. Child Care Health Dev. 2021;47:40–6. 33016377 10.1111/cch.12811

[25] Romisher R, Hill D, Cong X. Neonatal abstinence syndrome: exploring nurses’ attitudes, knowledge, and practice. Adv Neonatal Care. 2018;18:E3–e11. 29360671 10.1097/ANC.0000000000000462

[26] Dahl RA, Vakkalanka JP, Harland KK, Radke J. Investigating healthcare provider bias toward patients who use drugs using a survey-based implicit association test: pilot study. J Addiction Med. 2022;16:557–62. 10.1097/ADM.0000000000000970 PMC9537726 36201677

[27] Weber A, Miskle B, Lynch A, Arndt S, Acion L. Substance use in pregnancy: Identifying stigma and improving care. Subst Abus Rehabil. 2021;12:105–21. 10.2147/SAR.S319180 PMC8627324 34849047

[28] Schiff DM, Stoltman JJK, Nielsen TC, Myers S, Nolan M, Terplan M, et al. Assessing Stigma towards substance use in pregnancy: a randomized study testing the impact of stigmatizing language and type of opioid use on attitudes toward mothers with opioid use disorder. J Addict Med. 2022;16:77–83. 33758119 10.1097/ADM.0000000000000832 PMC8443692

[29] Substance use during pregnancy. New York, NY: The Guttmacher Institute, January 2022. (https://www.guttmacher.org/statepolicy/explore/substance-use-during-pregnancy).

[30] Faherty LJ, Stein BD, Terplan M. Consensus guidelines and state policies: the gap between principle and practice at the intersection of substance use and pregnancy. Am J Obstet Gynecol MFM. 2020;2:100137. 33089133 10.1016/j.ajogmf.2020.100137 PMC7571448

