# Supplementary material for: Prospective observational study quantifying maternal-fetal fentanyl transmission in epidural analgesia infusion using umbilical cord blood and neonatal meconium
Source: J Perinatol. 2025 Sep 18;46(4):563–8. doi: 10.1038/s41372-025-02416-9 (PMC13121003; doi:10.1038/s41372-025-02416-9)
Supplement: Supplementary file 1 — Supplemental Table 1 [file 41372_2025_2416_MOESM1_ESM.docx]

**Table S1**: Psychometric properties of varying thresholds of maternal epidural duration for the prediction of fentanyl concentration in meconium >0.05 ng/g

| Epidural Duration | Sensitivity | Specificity | PPV | NPV |
| --- | --- | --- | --- | --- |
| >5 hours  >4 hours  >3 hours | 64.9% (150/231)  73.6% (170/231)  84.0% (194/231) | 74.3% (26/35)  68.6% (24/35)  62.9% (22/35) | 94.3% (150/159)  93.9% (170/181)  93.7% (194/207) | 24.3% (26/107)  28.2% (24/85)  37.3% (22/59) |
